# The Dumb Speak

**Published:** 1871-09

**Authors:** 


### "THE DUMB SPEAK,"

Was considered a miracle in the olden time ; but it may be well for our readers to know that persons who were born dumb are learned to talk at the Clark Institution at Northampton, Mass., under the care of Miss Rogers, who receives the children of the poor and rich alike, to pay according to their means, the only requisite being a capacity to learn.

Deafness and dumbness, inability to hear or speak, often go together. An interesting case is given of a noble lady of this class, whose first-born was asleep one day, when she was seen by an unobserved person to enter the nursery on tip-toe, cautiously, stealthily; when she reached the bedside, she drew a large stone from under her shawl, raised it steadily, and dashed it — not on the baby's head, but on the floor. The little sleeper started, and the mother fell on her knees in tears of gratitude that her darling was not born deaf.

